# Supplementary material for: Verification of Footwear Effects on a Foot Deformation Approach for Estimating Ground Reaction Forces and Moments
Source: Sensors (Basel). 2025 Jun 13;25(12):3705. doi: 10.3390/s25123705 (PMC12197032; doi:10.3390/s25123705)
Supplement: Supplementary file 1 [file sensors-25-03705-s001.zip › S_figure.pdf]

Supplementary File S1: Time-series plots of the prediction results for slow and fast walking speeds

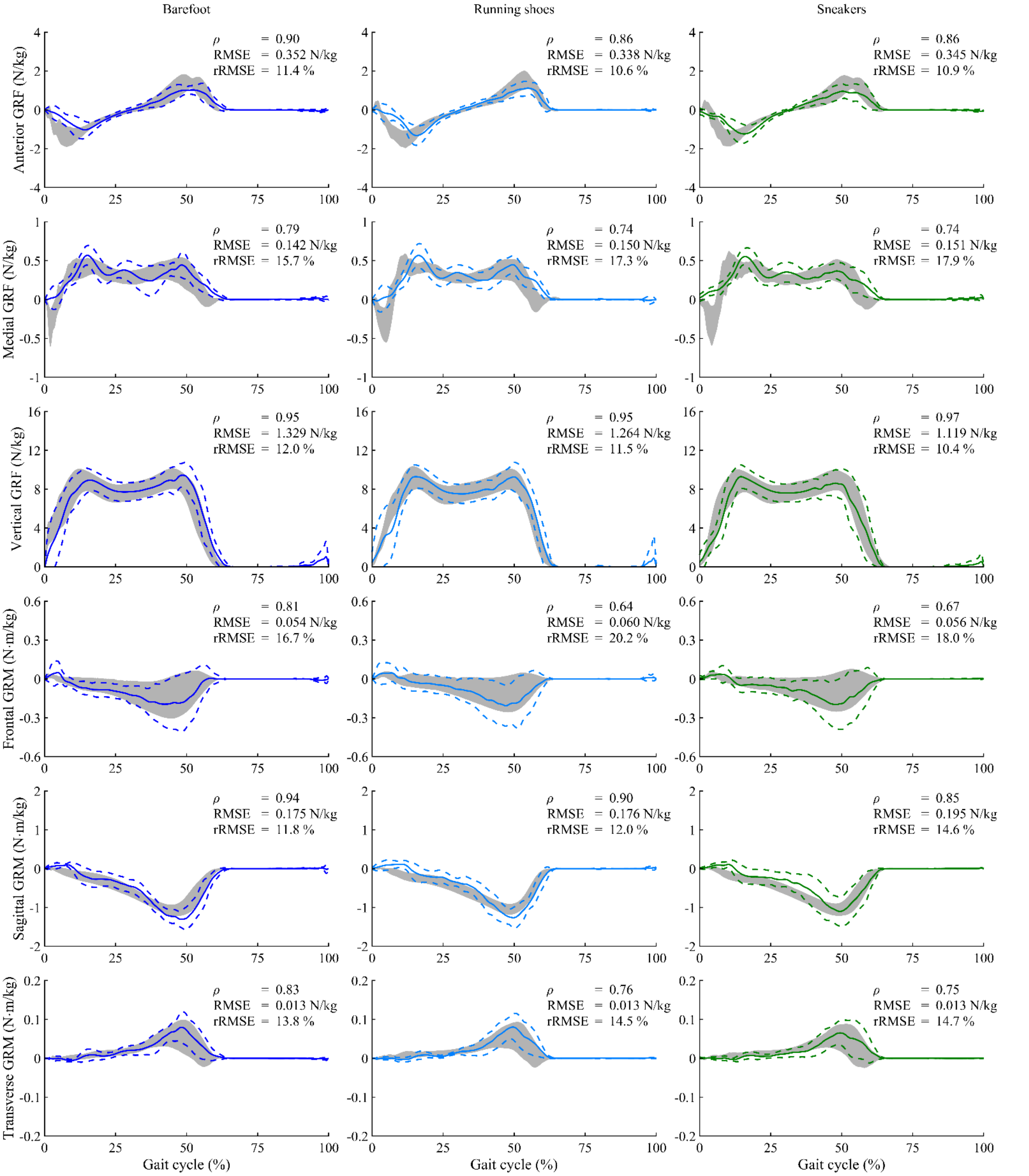

Figure S1. Ground reaction forces (GRFs) and moments (GRMs), normalized to the body mass of participants, during slow walking speed under three footwear conditions. Solid and dashed lines represent the predicted means and standard deviations for all participants, while gray shading indicates the measured values by the force plate. Each graph includes the Pearson's correlation coefficient (ρ), root-mean-square error (RMSE) and the relative RMSE (rRMSE). The positive directions of GRFs are defined along the anterior, medial, and upward axes, and GRMs are represented as moments around these axes.

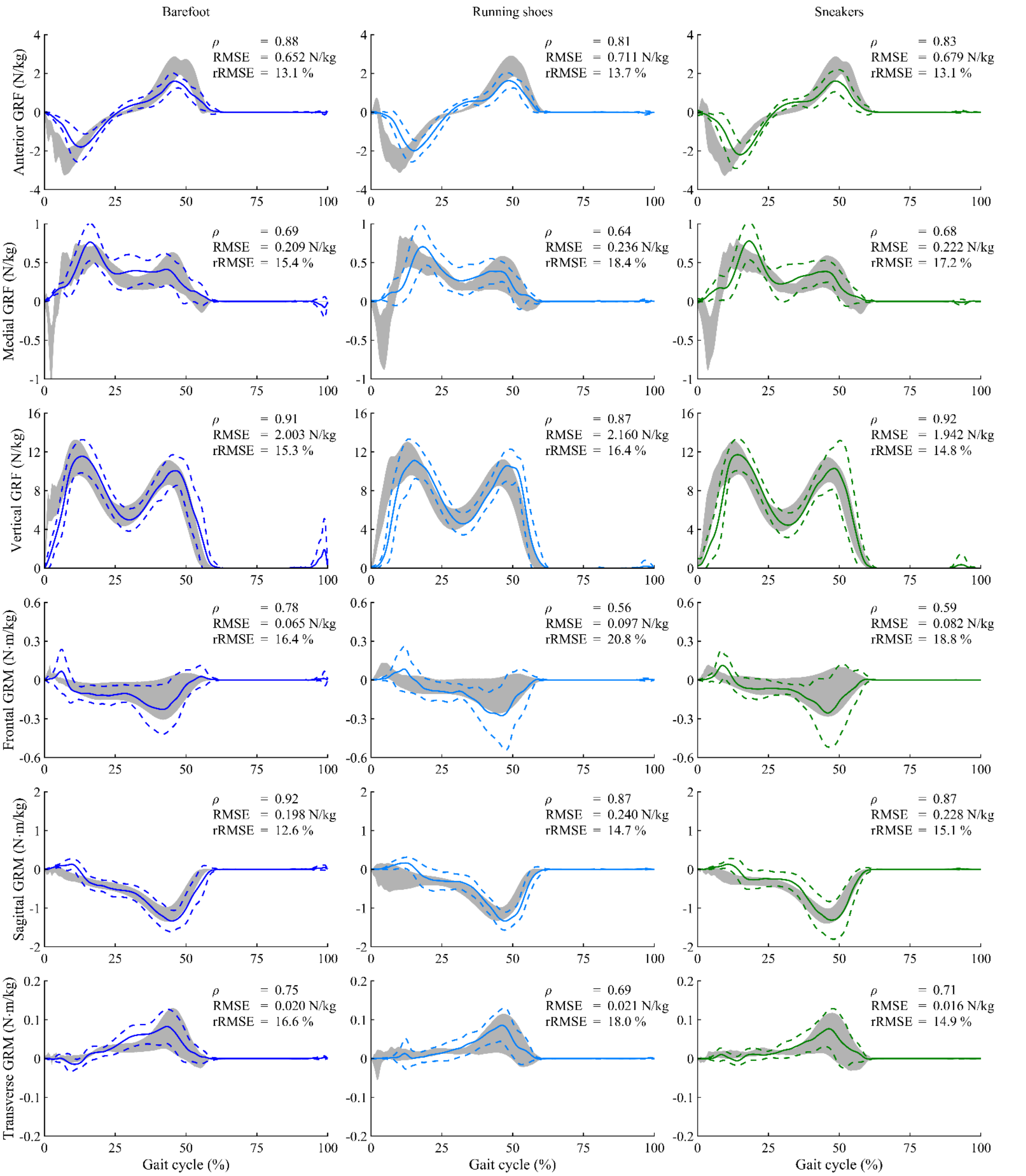

Figure S2. Ground reaction forces (GRFs) and moments (GRMs), normalized to the body mass of participants, during fast walking speed under three footwear conditions. Solid and dashed lines represent the predicted means and standard deviations for all participants, while gray shading indicates the measured values by the force plate. Each graph includes the Pearson's correlation coefficient (ρ), root-mean-square error (RMSE) and the relative RMSE (rRMSE). The positive directions of GRFs are defined along the anterior, medial, and upward axes, and GRMs are represented as moments around these axes.
