# Supplementary material for: Verification of Footwear Effects on a Foot Deformation Approach for Estimating Ground Reaction Forces and Moments
Source: Sensors (Basel). 2025 Jun 13;25(12):3705. doi: 10.3390/s25123705 (PMC12197032; doi:10.3390/s25123705)
Supplement: Supplementary file 1 [file sensors-25-03705-s001.zip › S_table.pdf]

Supplementary File S2: Prediction Accuracy for all experimental conditions

Table S1. Root-mean-square errors (RMSEs) and relative RMSEs (rRMSEs) in ground reaction forces (GRFs) and moments (GRMs) for all experimental conditions.

Footwear	Barefoot			Running shoes			Sneakers		
Speed	Slow	Normal	Fast	Slow	Normal	Fast	Slow	Normal	Fast
RMSE (N/kg or N▪m/kg)[1]									
Ant. GRF	0.352 (0.097)	0.452 (0.118)	0.652 (0.136)	0.338 (0.070)	0.462 (0.105)	0.711 (0.182)	0.345 (0.089)	0.426 (0.091)	0.679 (0.109)
Med. GRF	0.142 (0.025)	0.147 (0.039)	0.209 (0.032)	0.150 (0.036)	0.167 (0.029)	0.236 (0.061)	0.151 (0.026)	0.161 (0.032)	0.222 (0.047)
Ver. GRF	1.329 (0.418)	1.646 (0.422)	2.003 (0.484)	1.264 (0.530)	1.468 (0.315)	2.160 (1.039)	1.119 (0.379)	1.411 (0.457)	1.942 (0.334)
Fro. GRM	0.054 (0.018)	0.056 (0.023)	0.065 (0.022)	0.060 (0.026)	0.065 (0.023)	0.097 (0.037)	0.056 (0.018)	0.064 (0.016)	0.082 (0.027)
Sag. GRM	0.175 (0.032)	0.200 (0.045)	0.198 (0.030)	0.176 (0.037)	0.203 (0.033)	0.240 (0.099)	0.195 (0.055)	0.204 (0.045)	0.228 (0.086)
Tra. GRM	0.013 (0.003)	0.013 (0.004)	0.020 (0.005)	0.013 (0.004)	0.014 (0.003)	0.021 (0.008)	0.013 (0.003)	0.014 (0.003)	0.016 (0.004)
rRMSE (%)									
Ant. GRF	11.4 (1.7)	11.8 (1.9)	13.1 (1.7)	10.6 (2.5)	11.5 (1.5)	13.7 (2.4)	10.9 (1.9)	11.0 (1.2)	13.1 (2.6)
Med. GRF	15.7 (2.5)	14.7 (3.7)	15.4 (3.0)	17.3 (3.1)	16.9 (2.0)	18.4 (2.4)	17.9 (2.9)	17.1 (2.1)	17.2 (2.9)
Ver. GRF	12.0 (3.6)	14.3 (3.6)	15.3 (3.1)	11.5 (5.0)	12.6 (2.6)	16.4 (7.6)	10.4 (3.5)	12.2 (4.1)	14.8 (3.0)
Fro. GRM	16.7 (4.3)	17.2 (4.8)	16.4 (3.9)	20.2 (7.4)	18.8 (4.8)	20.8 (4.8)	18.0 (5.2)	18.6 (5.2)	18.8 (6.3)
Sag. GRM	11.8 (2.4)	13.1 (3.5)	12.6 (2.6)	12.0 (2.2)	13.4 (1.8)	14.7 (5.1)	14.6 (7.7)	14.1 (6.8)	15.1 (10.3)
Tra. GRM	13.8 (6.2)	12.5 (5.6)	16.6 (5.5)	14.5 (5.7)	13.7 (4.0)	18.0 (7.0)	14.7 (3.6)	13.4 (3.1)	14.9 (5.9)

Note: The average and standard deviations for all participants are displayed.

[1] GRFs are displayed in N/kg, and GRMs are displayed in N▪m/kg
